# A Mystery Unraveled: Essentiality of RNase III in *Bacillus subtilis* Is Caused by Resident Prophages

**DOI:** 10.1371/journal.pgen.1003199

**Published:** 2012-12-27

**Authors:** Fabian M. Commichau, Jörg Stülke

**Affiliations:** Department of General Microbiology, Georg-August-Universität Göttingen, Göttingen, Germany; University of Geneva Medical School, Switzerland

One of the key issues in our understanding of life is the study of the essential set of genes and proteins that make up a living cell and a living organism. Genome-wide studies have identified between 270 and 650 essential genes in bacteria and about 900 essential genes in yeast [1]. Once the essential gene set is known, questions are raised as to why these genes are essential and which important functions the encoded proteins fulfil. The issue is complicated by the fact that essential functions may be carried out by pairs of homologous genes that functionally replace each other and by convergent metabolic functions of non-homologous proteins [2], [3].

Comparative analyses of the essential gene sets of different bacteria have revealed a significant set of genes that is essential in all bacteria studied so far. These genes can be referred to as obligatory essential genes. In contrast, facultative essential genes are essential in one organism but may be non-essential or even absent in other organisms, related or unrelated. It is obvious that the obligatory essential genes encode proteins that fulfil the most important housekeeping functions, such as the processing of the genetic information. Indeed, many proteins involved in DNA replication, transcription and translation are conserved and essential in all bacteria.

## Essential RNases

Several RNases are essential in many bacteria. Most prominently, RNase E, the paradigm of a key enzyme for bacterial RNA degradation, is essential in *Escherichia coli*. RNase E organizes a protein complex, the RNA degradosome, but none of the other degradosome components nor the corresponding scaffold region of RNase E are essential. The reason for the essentiality of this protein has remained enigmatic [4]. In *Bacillus subtilis*, five RNases are essential. Two of them, RNase P and RNase Z, are required for the maturation of tRNA [5]. For the remaining essential RNases, III, J1 and Y, the reason for the essentiality is not so obvious. RNase Y was first identified as a potential interaction partner of the essential glycolytic enzymes enolase and phosphofructokinase. Recently, this RNase was proposed to be the functional equivalent of *E. coli* RNase E [6], [7]. Recent transcriptome studies failed to provide a clear explanation for the essential nature of the endoribonuclease Y and the exoribonuclease J1; however, several essential genes, among them those encoding aminoacyl-tRNA synthetases, enzymes of cytochrome c biogenesis, and the essential subunit of pyruvate dehydrogenase, are less expressed if RNase Y is limiting [8]–[11].

## A Protective Function for RNase III in *B. subtilis*

Interestingly, RNases III and Y are essential in *B. subtilis*, whereas they are non-essential in other related Gram-positive bacteria such as *Staphylococcus aureus* and *Streptococcus pyogenes* [11]–[14]. As discussed above, this facultative essentiality suggests that these RNases are required for the protection of the cell against toxic molecules or for other specific functions in the context of the *B. subtilis* cell. For RNase III, the existence of suppressor mutations allowing the deletion of the *rnc* gene was reported, suggesting that RNase III has a protective function [15]. In this issue of *PLOS Genetics*, Durand et al. [16] identify this essential function of RNase III. In their previous transcriptome analysis, the authors observed that depletion of RNase III resulted in the accumulation of toxin-encoding mRNAs [10]. Based on this observation, they have now performed a series of elegant genetic experiments to demonstrate that RNase III is indeed required for the degradation of the mRNAs of two toxin genes, *txpA* and *yonT*. These toxin genes are parts of a cryptic prophage, the skin element, and of the prophage SPβ, respectively. Once the *txpA* and *yonT* toxin genes are expressed, they can harm their own cell since the two mRNAs have the strongest ribosomal binding sites found in *B. subtilis*, suggesting that they are very efficiently translated to toxin protein [17]. The expression of these type I toxin/antitoxin systems is controlled by base-pairing with the specific antisense RNAs *ratA* and anti-*yonT* that form hybrids with the *txpA* and *yonT* toxin mRNAs, respectively. Biochemical experiments presented by Durand et al. [16] show that these base-paired RNA hybrids are subject to degradation by the activity of the double strand–specific endonuclease RNase III.

**Figure 1:**
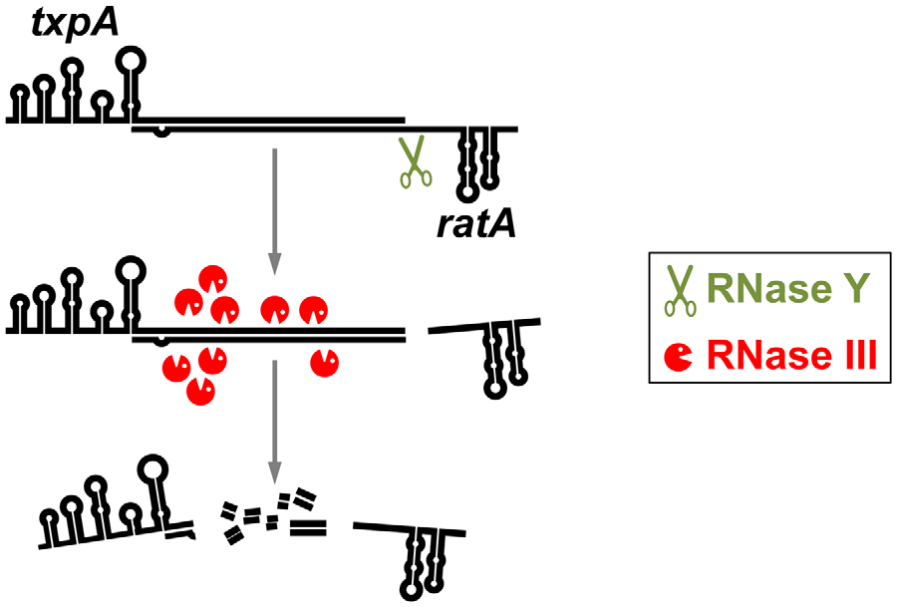
Degradation of a phage-encoded toxin mRNA in *B. subtilis*. The toxin-encoding mRNA *txpA* is degraded by the combined action of the antisense RNA *ratA* and the double strand–specific endonuclease RNase III. The *txpA–ratA* RNA hybrid may be destabilized due to prior processing by the essential RNase Y.

In the case of *txpA*, which has been studied down to the molecular details by Durand et al. [16], there is a 15-fold excess of the *ratA* RNA as compared to the *txpA* mRNA. This strong excess ensures that the *txpA* mRNA is always bound by the *ratA* RNA and thus targeted for degradation. Indeed, the absence of the *ratA* RNA results in a substantial stabilization of the *txpA* mRNA. This accumulation of *txpA* mRNA can only be tolerated if the mRNA cannot be translated due to a mutation of the start codon. The double-stranded *txpA* mRNA–*ratA* RNA hybrid molecule is cleaved in vitro by RNase III at multiple sites, resulting in the inactivation of the *txpA* message (see Figure 1). In addition, RNase Y was found to have a major cleavage site at a single-stranded region of the *ratA* RNA that is immediately adjacent to the double-stranded part of the *ratA* RNA. In consequence, cleavage of the *ratA* RNA by RNase Y results in a trimming of the end of the double-stranded RNA molecule, and in a destabilization of the *ratA* RNA, both in vivo and in vitro. It is tempting to speculate that the trimming of the *txpA* mRNA–*ratA* RNA hybrid molecule facilitates recognition of and/or access to the complex by RNase III (see Figure 1). Indeed, the absence of RNase Y results in a duplication of the *txpA* mRNA half-life from 1.1 to 2.4 minutes. It should, however, be noted that the depletion of RNase III increases the half-life of the *txpA* mRNA to more than 20 minutes. Thus, the major role of RNase Y might be the fine-tuning of the *ratA–txpA* RNA ratio. Similar to the *txpA*/*ratA* system, RNase III cleaves the hybrids between *yonT*/as-*yonT* and *bsrG*/as-*bsrG*, resulting in the degradation of the toxin mRNAs [16], [18]. Interestingly, the *bsrG*/as-*bsrG* duplex is cleaved by RNase III downstream of the toxin open reading frame, leaving the question of how RNase III might affect the control of toxin synthesis [18].

The current study by Durand et al. [16] explains why RNase III is essential in *B. subtilis*, whereas it is dispensable in most other bacteria. It is tempting to speculate that this facultative essentiality of RNase III (as well as of other RNases such as RNase Y) is directly coupled to the presence of toxin systems in the genomes where the RNases are essential. However, due to the experimental investigation of only few laboratory strains and the genomic variability of bacteria with non-essential RNases III and Y (especially with respect to the presence of prophages), no clear statement about such a correlation is possible at the moment. From an evolutionary point of view, a next interesting question would address the reason for the persistence of several prophages and sequences derived from prophage in the *B. subtilis* genome even though these sequences do not provide any obvious selective advantage to the cell.
